# Measuring children’s sustained selective attention and working memory: validity of new minimally linguistic tasks

**DOI:** 10.3758/s13428-023-02078-5

**Published:** 2023-02-17

**Authors:** Kerry Danahy Ebert, Giang T. Pham, Sophie Levi, Benjamin Eisenreich

**Affiliations:** 1 Department of Speech-Language-Hearing Sciences, University of Minnesota Twin-Cities, 115 Shevlin Hall, 164 Pillsbury Dr. SE, Minneapolis, MN 55455 USA; 2 School of Speech, Language, and Hearing Sciences, San Diego State University, San Diego, CA USA; 3 Joint Doctoral Program in Language and Communicative Disorders, San Diego State University and University of California San Diego, San Diego, CA USA; 4 Center for Applied & Translational Sensory Science, University of Minnesota-Twin Cities, Minneapolis, MN USA

**Keywords:** Cognition, Development, Assessment

## Abstract

This study introduces visual tasks using nonlinguistic stimuli that measure sustained selective attention (SSA) and working memory (WM), two constructs foundational to learning and associated with developmental disorders in children. Using an argument-based approach to validation, we examine whether each task (a) measures distinct constructs, (b) shows internal consistency, (c) captures a range of performance, and (d) relates to development as indexed by age. Participants included 71 children, ages 4–10, of whom 12 had parental concern for language/learning. The SSA task presented spatial locations within a long and uninteresting task, following the continuous performance task paradigm. The WM task presented paired location sequences of increasing length, incorporating key elements of the *n*-back and complex span paradigms. Controlling for age, tasks were found to be minimally associated with each other (*r* = .26), suggesting related but distinct constructs. Internal consistency was high, with split-half reliability of .94 (SSA) and .92 (WM); the stability of these estimates was supported by bootstrapping simulations. Task performance was evenly distributed, with minimal floor or ceiling effects within this age range. Performance was positively related to age (SSA *r* = .49; WM *r* = .53). Exploratory correlations with a measure of parental concern were significant for SSA but not WM. The results show that these new tasks can be used to measure children’s SSA and WM in a visual domain with minimal linguistic influence. These tasks capture developmental changes in the early school years. Further investigation can examine their utility for classifying children with developmental disorders.

Attention and memory are broad cognitive processes that are foundational to learning new information. Within each of these processes, multiple subcomponents have been identified that may contribute uniquely to learning. To fully capture attention and memory in children, reliable and valid measures across the range of subcomponents are needed. The present study focuses on two specific subcomponents—sustained selective attention and working memory—that have been linked to developmental disorders that affect learning, including developmental language disorder (DLD; e.g., Ebert & Kohnert, 2011; Gillam et al., 2021; Smolak et al., 2020; Vugs et al., 2013) and attention deficit hyperactivity disorder (ADHD; e.g., Heaton et al., 2001; Huang-Pollock et al., 2012; Martinussen et al., 2005).

This study describes two new tasks designed to measure sustained selective attention and working memory within early school-aged children. The tasks were designed to capture the development of these constructs and to identify weaknesses that may relate to developmental disorders. The automated, computer-based tasks use nonlinguistic stimuli to avoid disadvantaging children with different language-learning experiences (e.g., exposure to languages other than English) or linguistic abilities (e.g., DLD). In the sections that follow, we define the constructs of sustained selective attention and working memory and describe common approaches to assessing these constructs in children. We also briefly review literature linking each construct to developmental disorders including DLD and ADHD. These sections lay the foundation for the evaluation of these tasks using an argument-based approach to validity (Kane, 2013, 2015).

## Sustained selective attention

Attention refers to a set of cognitive processes that determine which stimuli are selected for further processing. Component models of attention (e.g., Gomes et al., 2000; Mirsky et al., 1991; Petersen & Posner, 2012) explain the complex attentional system by dividing it into separable pieces. Within these models, both *selective* and *sustained* attention are common components. Selective attention is the subconscious process of focusing on certain information and ignoring other information, while sustained attention refers to maintaining this focus over an extended period of time. Because sustained attention inherently involves selective attention, it is more accurately termed sustained selective attention.

Sustained selective attention is commonly measured using the continuous performance task (CPT), also known as a go/no-go task (Riccio et al., 2002). In this paradigm, the participant is asked to monitor a stream of stimuli and respond to a target (“go”) stimulus while ignoring non-target (“no-go”) stimuli. To assess sustained selective attention, the task must be sufficiently long to tax vigilance over time (Riccio et al., 2002). Performance can be measured in terms of accuracy or reaction time to target stimuli.

Despite the apparent simplicity of the task, the design of a CPT includes several parameters that may influence performance (Corkum & Siegel, 1993; Roebuck et al., 2016). One such parameter is the type of stimuli. Both auditory and visual stimuli are possible; for example, the participant might view a series of shapes or letters on a screen or might listen to a series of tones or words. Within each modality, the processing load imposed by specific types of stimuli can vary and may drive performance (Roebuck et al., 2016). Another parameter to consider is the extent to which stimuli possess linguistic content. Stimuli that explicitly engage linguistic processing (e.g., words) may confound the assessment of attention with language knowledge, whereas nonlinguistic stimuli can provide a purer measure of attention alone (Ebert & Kohnert, 2011; Kaushanskaya et al., 2017).

In addition to the type of stimuli, CPTs vary in other parameters, including the proportion of stimuli that are targets, the duration of stimuli and the intervals between them, the number of distinct non-target stimuli, and the overall task duration. It is generally accepted that targets should make up a small proportion of the stimuli in order to tap into sustained selective attention; when targets appear too frequently, the task may emphasize inhibition over vigilance (Riccio et al., 2002). However, there is no single accepted value for this parameter, and sustained selective attention tasks used with children vary. For example, Ebert and Kohnert’s (2011) review of CPT studies in children with DLD found the proportion of targets to vary from 10% to 50%; Corkum and Siegel’s (1993) review of CPT studies in children with ADHD found the proportion of targets varied from 10% to 25%. Similarly, though it is accepted that a relatively long task is needed to assess sustained selective attention, there is no clear consensus on the required length; tasks reviewed in Corkum and Siegel (1993) ranged from 5 to 15 minutes, and a commercially available CPT marketed for the purpose of identifying ADHD (Test of Variables of Attention; Greenberg et al., 2020) runs for 22 minutes. In the absence of clear criteria for effective CPT design, it is important to clearly report all task parameters.

## Child-internal factors affecting sustained selective attention

Valid assessments of sustained selective attention for children will capture individual differences in skill level for the underlying construct. In other words, “individual differences” refers to stable differences in attentional capacity between children, rather than temporary differences resulting from factors such as fatigue (e.g., Barry et al., 2005). Two key predictors of individual differences in sustained selective attention are chronological age and developmental disorders. There have been varied findings on the trajectory of sustained selective attention development (e.g., timing of plateaus vs. rapid growth). For example, in a sample of 57 Australian children Betts et al. (2006) found rapid increases in attention indices from ages 5–6 years to ages 8–9 years and suggest a plateau in sustained selective attention development after age 10. Lin et al. (1999) tested 341 Taiwanese children aged 6 through 15 years using a CPT and reported a quadratic relationship between performance and age, with rapid development from age 6 through 10 years on most measures. In contrast, Kanaka et al. (2008) reported that the ability to discriminate targets within a CPT begins to plateau at age 8 years; their conclusions were based on a sample of 541 Japanese girls. Subtle differences in findings across these studies may relate to variations in task parameters, research settings, and sample characteristics. Ultimately, even though the timing and shape of change remain open questions, there is consensus that sustained selective attention increases during the primary school years.

Weaknesses in sustained selective attention have also been linked to developmental disorders that affect learning. We focus specifically here on ADHD and DLD because of the robust literature linking attention and memory weaknesses to these disorders and the potential for measures of attention and memory weaknesses to contribute to diagnostic decisions. ADHD, a disorder defined by clinical deficits in attention, is unsurprisingly associated with poorer sustained selective attention (e.g., Heaton et al., 2001). Meta-analytic synthesis of studies comparing the CPT performance of children with and without ADHD has yielded significant between-group differences with moderate effect sizes (Huang-Pollock et al., 2012). However, weaknesses in sustained selective attention are not restricted to children diagnosed with ADHD. In particular, a substantial body of literature on children with DLD (a disorder defined by impairments in language without a known biomedical condition; Bishop et al., 2017) supports the presence of subtle impairments in sustained selective attention within this population (Ebert & Kohnert, 2011; Ebert et al., 2019; Smolak et al., 2020). Like the literature on ADHD, meta-analytic synthesis of studies comparing the CPT performance of children with and without DLD (Ebert & Kohnert, 2011) yielded significant differences between groups. Moreover, performance deficits remained when task stimuli were nonlinguistic and when children with comorbid ADHD were excluded from samples, supporting the conclusion that sustained selective attention deficits are associated specifically with DLD. Although there is debate regarding the exact role of attention skills in DLD, it is possible that weaknesses in sustained selective attention result in children with DLD missing relevant but less salient portions of linguistic input (see Ebert et al., 2019; Smolak et al., 2020 for more discussion).

## Working memory

Memory is commonly defined as a cognitive system devoted to storing and recalling relevant information; within this system, working memory serves to maintain the information needed to perform current cognitive operations. Although foundational models of working memory posited separate modules for different types of information (Baddeley, 1992), more recent conceptualizations construe working memory as a more general system that operates across all types of input (e.g., Kane et al., 2004). Most models of working memory include a storage component along with executive control processes that select and maintain relevant information within the storage (Miyake & Shah, 1999). Both storage and executive controlled processing are governed by capacity limitations, potentially with a tradeoff between them.

Working memory has been traditionally measured using complex span tasks, in which a participant is asked to store and later recall a set of items while simultaneously performing another action (e.g., Danahy et al., 2007; Daneman & Carpenter, 1980). For example, Daneman and Carpenter (1980) created the reading span task, in which a participant reads a series of sentences aloud and is required to retain the last word in each one. A critical aspect of complex span tasks is that the number of items to be remembered increases adaptively with performance. In other words, the task continues until the sets become too large for a participant to retain.

Numerous studies have employed complex span tasks with children, with the varied span tasks integrating different types of stimuli and cognitive operations (e.g., Conway et al., 2005; Gordon et al., 2020). For example, counting span (e.g., Danahy et al., 2007) does not require reading; rather children count the number of dots appearing on a screen aloud and must retain the totals across a series of screens. Span tasks typically advance when the participant is successful and discontinue when they are not, though specific criteria for “success” may be as low as 50% of trials correct (Gordon et al., 2020). Conway et al. (2005) recommend a set size of two to five items as optimal for eliciting individual differences and avoiding ceiling effects.

Although complex span tasks remain a viable paradigm for working memory assessment, participant response formats within these tasks can vary (e.g., different types of spoken responses), and often pose a disadvantage for neuroimaging studies. During the past two decades, an alternative paradigm—the *n*-back task—has become a prominent approach to measuring this construct (Kane et al., 2007; Pelegrina et al., 2015). In the *n*-back paradigm, the participant must recognize whether a stimulus matches a previous stimulus. Task complexity is often controlled by increasing the number of items in between the two stimuli to be compared: in a *1-*back task, the participant compares the current stimulus to the one presented immediately prior; in a *2*-back, the current stimulus is compared to one presented two positions back (i.e., with an intervening item).

The *n*-back paradigm was originally implemented with adult participants and relatively few investigations have been conducted with children younger than 7 years (Yaple & Arsalidou, 2018). Younger children may have particular difficulty with task complexities beyond the *1*-back level (Pelegrina et al., 2015). In the somewhat limited literature on *n-*back tasks with children, stimuli have included spatial locations, pictures of common nouns, semantic categories, letters, or words (Ciesielski et al., 2006; Pelegrina et al., 2015; Yang & Gray, 2017). Other task parameters—such as the proportion of matching versus nonmatching stimuli, the length of time available for a response, and the duration of the stimuli—are likely to influence performance, but analyses of these parameters with young children are not yet available.

The present study introduces a working memory task that uses an n-back paradigm, namely a 1-back because young children have difficulty beyond this level (Pelegrina et al., 2015). To further tailor the task to a child audience, this new task also has adaptive features from complex span, namely: criteria for advancing task difficulty (i.e., increasing the number of items to be remembered), criteria for discontinuing the task, and the maximum size of the set of items to be remembered. Together, the 1-back paradigm with these adaptive features from complex span contribute to an overall shorter length of the task. Task length is critical to consider for child populations, particularly to measure working memory in isolation rather than tapping into working memory and attention.

## Child-internal factors affecting working memory

As with sustained selective attention, working memory is influenced by age as well as the presence of developmental disorders (and, as with sustained selective attention, performance on working memory tasks may be affected by transient internal states as well as external conditions, but we focus here on predictors of individual differences). It is clear that working memory capacities increase in children through the primary school years. For example, Gathercole et al. (2004) documented steady improvement within the 4- to 10-year-old age range on working memory tasks across a variety of stimulus types. Increases in knowledge (i.e., familiarity with stimuli) have been proposed as an alternative explanation for these gains, but studies attempting to control for knowledge have continued to support working memory gains (Cowan, 2016). It should be noted that the majority of studies on working memory development in children have been conducted within the span paradigm rather than the *n*-back. For example, Peng and Fuchs (2016) conducted a meta-analysis of working memory skills in children with learning difficulties; of the 17 included studies, 15 utilized a complex span task and 0 utilized an *n*-back. However, initial data from Pelegrina et al. (2015) indicate that *n*-back tasks also demonstrate developmental changes, at least within the 7- to 13-year age range.

Working memory weaknesses are also robustly associated with developmental disorders. Working memory has been studied extensively in the context of ADHD; a 2005 meta-analysis (Martinussen et al., 2005) included 26 studies comparing children with ADHD to unaffected peers and concluded that children with ADHD demonstrate deficits in working memory that are unexplained by comorbid language disorder or by overall intellectual capabilities. Subsequent investigations have linked working memory specifically to the severity of ADHD symptoms and associated working memory development with symptom improvement (e.g., Karalunas et al., 2017).

Impairments in working memory have also been documented for children with DLD. Deficits are particularly pronounced on working memory tasks that use verbal material (e.g., Archibald & Gathercole, 2006), though such investigations may confound language and working memory deficits. However, a meta-analysis of 21 studies focused specifically on nonlinguistic, visuospatial working memory tasks (Vugs et al., 2013) found significant deficits in children with DLD in comparison to peers, with the severity of visuospatial working memory deficits linked to the severity of language impairment. A recent investigation of 234 school-age children with and without DLD found that working memory-related cognitive abilities, measured via a mix of linguistic and nonlinguistic tasks, explained 93% of the variance in language ability in the DLD group, underscoring the role of working memory in DLD (Gillam et al., 2021). As with sustained selective attention, explanations for the relationship between working memory abilities and language deficits in children with DLD vary. It is possible that decreased efficiency in processing information for working memory storage, decreased storage capacity, or both in combination impair the comprehension of linguistic input—an activity which taxes working memory—thus slowing language acquisition (see Gillam et al., 2021; Smolak et al., 2020; Vugs et al., 2013).

## Present study

The present study evaluates new tasks of sustained selective attention and working memory for children. These tasks contribute to existing measures in that they use nonlinguistic stimuli to reduce bias resulting from differences in language experiences or abilities, they are fully automated, and they are freely available (see Open Practices Statement). We also offer an evaluation of validity alongside the tasks, following an argument-based approach (Kane, 2013, 2015). The argument-based approach holds that the validation process needs to align with the potential uses of a new task. Thus, a validation argument specifies the proposed uses and interpretations of a task, and then the argument can be evaluated for its coherence, completeness, and plausibility. This approach contrasts with other validation approaches which seek to establish specific subtypes of validity but do not specify the relationship between those subtypes and the way the test scores will be used. In a systematic review, Lavery et al. (2020) identified 83 articles that have used an argument-based approach to validity. One of the key themes identified in their review is that argument-based validation—that is, the proposed uses and interpretations of an assessment—needs to be considered in its design and development. For example, an assessment that will be used to certify competency in an area of skill must be designed differently than an assessment designed to measure growth over time. In our case, we sought to develop tasks that could detect individual differences, particularly those related to age or developmental disorders, in two specific cognitive constructs.

The overall goal of this study is to introduce two new measures of cognitive processing using visual, nonlinguistic stimuli and to examine their validity for use with children. We created a sustained selective attention task within the CPT paradigm and a working memory task within the *n*-back paradigm that integrates elements of the span task as well. Then we administered the tasks to a sample of 4- to 10-year-old children (*n* = 71). We propose that the two tasks are valid measures of their respective constructs that can be used to assess individual differences in early school-age children and potentially detect weaknesses in these areas. In line with argument-based validity (Kane, 2013, 2015), we offer the following arguments and analyses to support task validity:

1. Each task measures its intended construct.

The creation of tasks within the CPT and *n*-back paradigms provides initial support for this argument. As previously described, these paradigms are derived from theories of sustained selective attention and working memory, respectively. To further support this argument, we will examine the correlation between scores on the two tasks. Because both sustained selective attention and working memory are components of an individual’s cognitive ability and both are measured here in the visual domain with nonlinguistic stimuli, we would expect a significant relationship between the tasks. However, inter-task correlations should be moderate at best, given that the intended constructs are distinct (Swank & Mullen, 2017).

2. Task scores provide consistent estimates of a child’s sustained selective attention and working memory.

To support this argument, we will examine internal consistency reliability, as measured by the correspondence between a child’s scores on one half of the task with the other half of the same task (see Analyses for additional details). Internal consistency reliability demonstrates the generalizability of task scores and is a necessary component of most validity arguments (Kane, 2015).

3. The tasks are sensitive to a range of abilities within the intended age range.

To support this argument, we will examine the distribution of scores within the 4- to 10-year-old age range included in this study. Minimal evidence of floor and ceiling effects as well as a continuous score distribution can provide support for this argument. If the tasks were too easy to measure higher abilities within this age range, ceiling effects would be expected; if the tasks were too difficult to measure lower abilities within this age range, floor effects would be expected.

4. Performance on the tasks relates to external measures of development.

To support this argument, we will examine relations between chronological age and task performance. We expect task performance to be associated with age because sustained selective attention and working memory should progress during the 4- to 10-year age range.

We also conduct an exploratory analysis of the relations between task performance and a broad measure of parental concern regarding the development of language and attention. The purpose of this analysis is to collect preliminary evidence of task suitability for detecting weaknesses in underlying sustained selective attention or working memory skills; positive associations between parental concern and task performance would provide preliminary support and encourage further exploration in this area.

## Methods

This study was approved by the Institutional Review Board at the University of Minnesota. Informed consent was obtained in writing from parents prior to participation and informed assent was obtained orally or in writing from children, consistent with their age. Testing was conducted within the University of Minnesota research building at the State Fair, immediately following parental consent. Upon completion of the study, families received a $10 gift card for their participation.

### Participants

The participant sample was recruited over 4 days at the Minnesota State Fair, a 12-day event that drew approximately 1.3 million visitors in 2021 (Minnesota State Agricultural Society, 2021). Families with children aged 4–10 years who were inside or near the University of Minnesota research building at the Fair were invited to participate. A total of 77 parents consented. Within this sample, three children were unwilling to participate and three children were deemed to be ineligible after analysis of the parent interview responses (*n* =2 reported a diagnosis of autism and *n* = 1 reported a previous traumatic brain injury). One additional child refused the sustained selective attention task only.

The final sample thus included 71 children (*n* = 70 for sustained selective attention) aged 4 years, 0 months, to 10 years, 11 months (M = 8.0 years, SD = 1.9 years). Thirty-seven children were male (per parent report) and 34 were female. Per parent report, all children demonstrated normal hearing and visual acuity (with or without corrective lenses). Children had no history of autism spectrum disorder, cognitive delay, traumatic seizure disorder, cerebral palsy, or any other condition that would cause language disorders. All parents reported that English was the primary language used in the home. For four children, an additional language was also reported to be used in the home; additional languages included Spanish, Bengali, Haitian Creole, and Hmong. The highest level of parental education ranged from a high school diploma to a doctorate degree, with a median level of bachelor’s degree within the sample.

### Child tasks

Children completed four tasks (including the working memory and sustained selective attention tasks) in approximately 25 minutes, with individual guidance and supervision from a research staff member. The two cognitive processing tasks were implemented in E-Prime 3.0 (Psychology Software Tools, 2018). Responses for both tasks were collected using a Chronos response box connected to a laptop via USB to record responses with millisecond accuracy (Psychology Software Tools, 2019). Both tasks begin with an instruction phase, in which the task is explained to the child with demonstration trials, followed by a practice phase, in which the child completes sample items and receives feedback. Both tasks contain the option to repeat the practice phase if the child shows inadequate accuracy on the practice items. Instructions to examiners indicate that practice should be repeated if the child’s accuracy is below approximately 70–75% in the practice phase (specifically, 7 of 10 practice trials in the sustained selective attention task and 3 of 4 trials in the working memory task); however, the instructions also provide latitude to examiners to repeat the practice phase if the child expresses confusion or exhibits difficulty despite high accuracy in practice.

### Sustained selective attention

In the sustained selective attention task, each trial presents a black square onscreen. The location of the square determines whether it is a target or non-target (distractor) stimulus. Following the go/no-go paradigm, children are asked to respond to the target stimuli via button press and to ignore the distractor stimuli. A relatively large number of trials (320) are presented to assess performance on an uninteresting task after an extended time. The task lasts approximately 8 minutes.

**Fig. 1:**
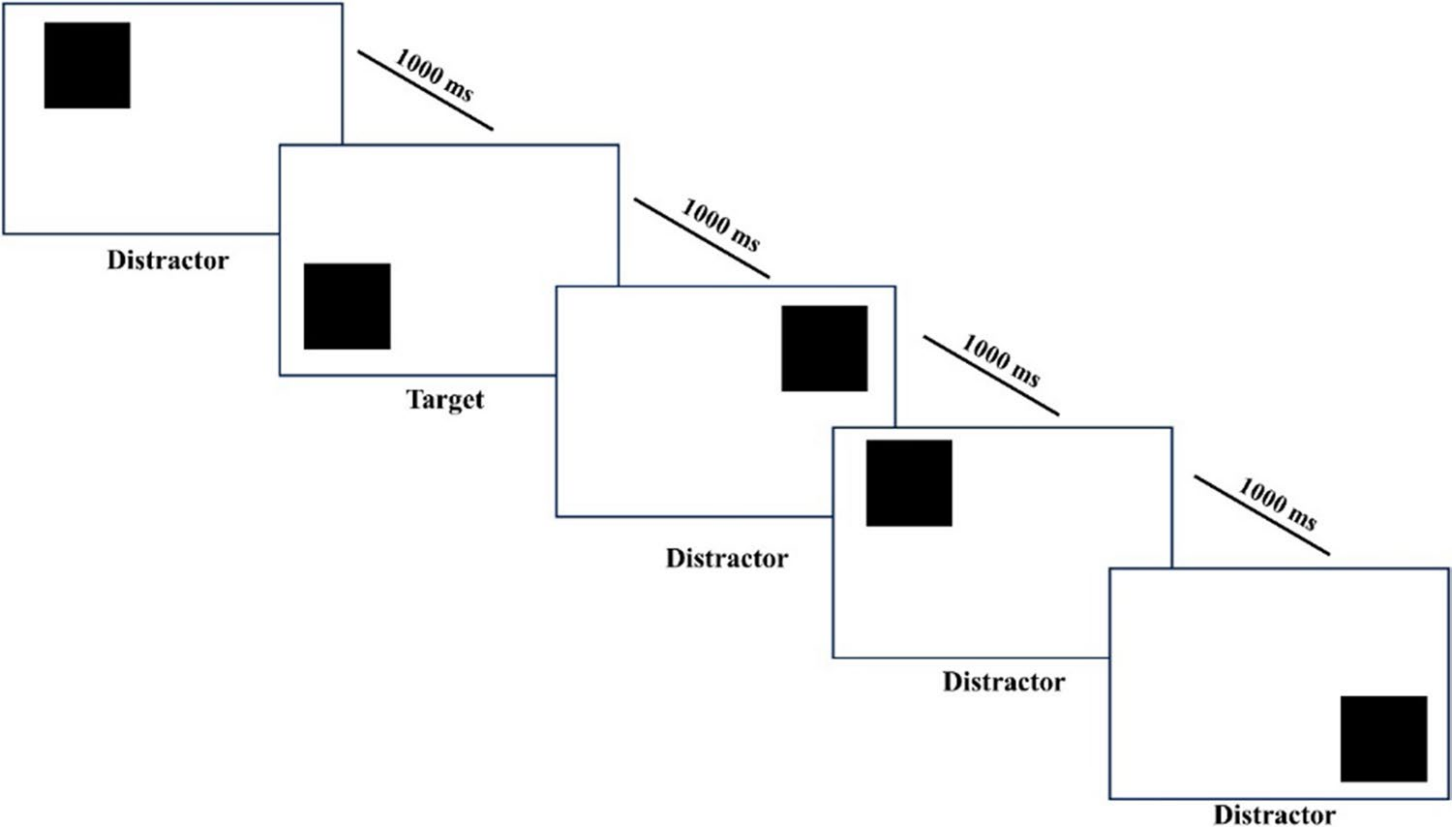
A sequence of five trials in the sustained selective attention task

Figure 1 shows a possible series of five trials for the sustained selective attention task. There are four possible locations for the squares: one per quadrant of the screen. The lower left quadrant was defined as the target location. Squares appeared in this location on 64 trials (20%), with the location for the remaining 256 trials (80%) distributed randomly across the other three quadrants (upper left, upper right, lower right). Each stimulus appeared for 400 milliseconds or until the response button was pressed, followed by a 1000-millisecond interstimulus interval.

The primary dependent variable for the sustained selective attention task is *d’* (d-prime; Macmillan & Creelman, 1991), a signal detection measure that combines accuracy on both target and distractor trials. *d’* is calculated as the difference between the *z-*transformed hit rate (i.e., proportion of targets on which the participant responds) and the *z-*transformed false alarm rate (i.e., the proportion of non-targets on which the participant responds).

$${d}^{\prime }=z\left(\frac{total\ hits}{number\ of\ targets}\right)-z\left(\frac{total\ false\ alarms}{number\ of\ non- targets}\right)$$

However, a perfect hit or false alarm rate is problematic because the *z*-transformation cannot be completed; several potential adjustments to the formula have been suggested. We followed a loglinear approach (Hautus, 1995) and added a proportional value to all cells to prevent perfect hit or false alarm rates:

$$d{\prime}_{adj}=z\left(\frac{total\ hits+ proportion\ of\ targets}{number\ of\ targets+2\ast proportion\ of\ targets}\right)-z\left(\frac{total\ false\ alarms+ proportion\ of\ false\ alarms}{number\ of\ non- targets+2\ast proportion\ of\ false\ alarms}\right)$$

A *d’*_*adj*_ value of 0 indicates an inability to differentiate between targets and non-targets, negative values indicate the participant is more likely to respond to a non-target than a target, and positive values indicate the participant is more likely to respond to a target than a non-target. Using the loglinear adjustment, the maximum *d’*_*adj*_ value on our task was 5.47.

### Working memory

In the working memory task, each trial presents two sequences of spatial locations. Children are asked to determine whether the pattern of spatial locations across the two sequences are the same or different. Phrased differently, children must determine if the stimulus (i.e., sequence of locations) matches the stimulus *1*-back, aligning this task with the *n*-back working memory paradigm. However, the task also retains an element of the span paradigm: stimulus complexity is increased in an adaptive fashion. After a block of four consecutive correct responses, one additional spatial location is added to each sequence within a trial.

**Fig. 2:**
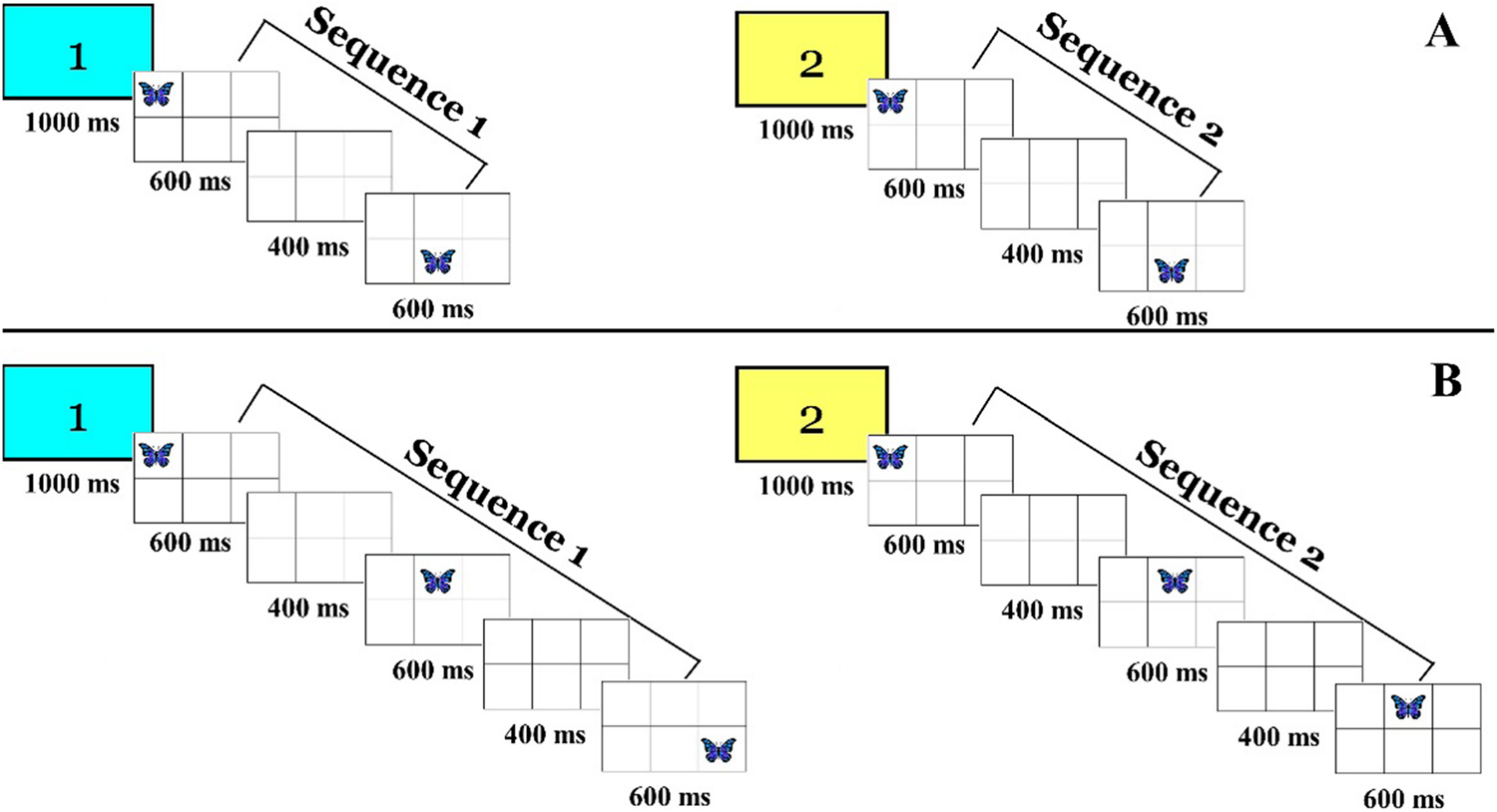
Sample trials in the working memory task. Panel A shows a sample trial with two locations per sequence and a matching (“same”) pair of sequences. Panel B shows a sample trial with three locations per sequence and a nonmatching (“different”) pair of sequences. Within each trial, sequence 1 is compared to sequence 2

Figure 2 illustrates sample trials in the task. Within each trial, a blue screen with a “1” is presented for 1000 milliseconds to indicate the beginning of the first sequence. Next, a six-square grid appears on the screen with an image of a butterfly in one of the six squares for 600 milliseconds, followed by a blank grid for 400 milliseconds and then the same grid with the butterfly in a different location for 600 milliseconds. The start of the second sequence is signaled by a yellow screen with a “2” on it, displayed for 1000 milliseconds, followed by two more butterfly locations with the same timing as above. The child then indicates by button press whether the two sequences of locations were the same or different.

**Fig. 3:**
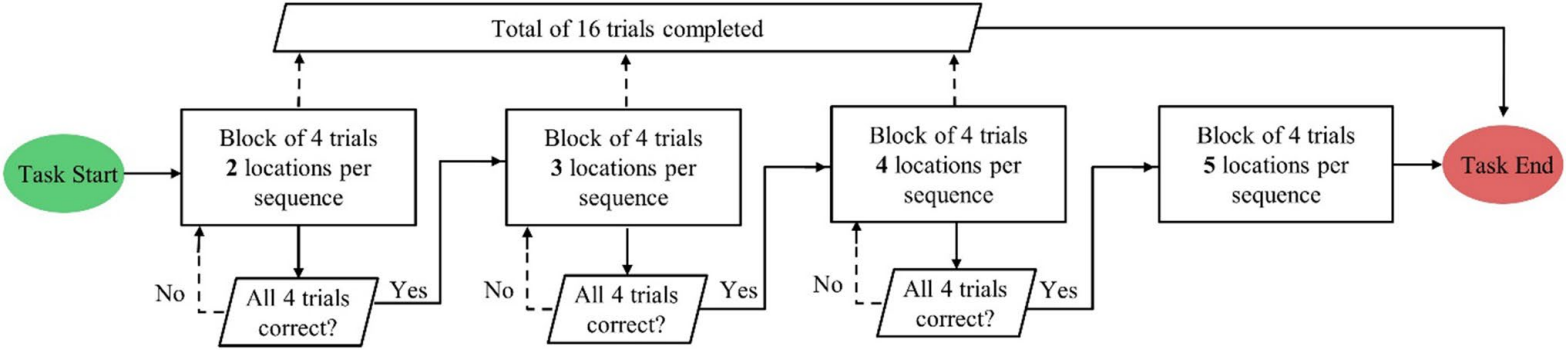
Flowchart of sequence length adaptation in the working memory task

The task includes four blocks of four trials (Fig. 3). In the first block, each sequence contains two locations. If all responses are correct in the first block, sequence length is increased to three locations in the next block. If one or more errors are made in the first block, the second block continues to contain two locations per sequence. This process is repeated for the third and fourth block, with sequence length increasing if all trials in a block are correct or remaining the same if there are one or more errors in the block.

For each sequence length, a set of 24 possible pairs of location sequences was generated, with half of the pairs the same and half different. On each trial, the task randomly selects one of the 24 pairs to be presented. Within the 12 possible sequence pairs that are different, the point at which it is possible to determine that the sequences are different is controlled to prevent the participant from needing to wait through many items after detecting the difference. More specifically, on the four- and five-item sequences, the location at which difference can be detected occurs only within the last three items. On the two- and three-item sequences, the location at which difference can be detected can occur in any position.

The primary dependent variable is an overall accuracy score, weighted by the sequence length for each correct item. The weighting system is shown in the following equation:

$${\displaystyle \begin{array}{c}\sum\limits_{n=1\kern1em }^n{x}_n\ast {w}_{i\kern0.5em }\\ {} where\kern0.5em x=\left\{\begin{array}{c}1\\ {}0\end{array}\right.\\ {} and\ {w}_i=\left\{\begin{array}{c}2\\ {}3\\ {}4\\ {}5\end{array}\right.\end{array}}$$

where subject response on each of the 16 trials x = (0, 1) and *w*_*i*_ is the sequence length for the item (2,3,4,5). For example: a participant who answers all four trials with two-item sequences correctly would be presented with three-item sequences; she answers two items in that block correctly (2 trials × 3-item sequence) and receives a second block of three-item sequences, in which she answers all four correctly (4 trials × 3-item sequence); she is then presented with a block of four-item sequences in which she answers one correctly (1 trial × 4-item sequence). The task stops after these 16 trials, and she earns a score of (4 × 2) + (2+4 × 3) + (1 × 4) = 30. The weighting system creates a maximum score of 56.

### Parent interviews

While children were completing the cognitive processing tasks, their parents were interviewed by a different research staff member. The interview established demographic characteristics, confirmed the absence of exclusionary conditions, and characterized the home language environment (e.g., number of languages spoken in the home). The interview questions also probed for parent concern about development.

In order to explore any potential relations with task performance, we opted to use responses to one question, “Are there any additional medical or developmental concerns for your child?” to identify participants with parental concerns about development. Parent responses to this question contained information not provided in other responses and supported the division of participants into two broad categories. Parent responses indicating concerns related to attention, language, reading, or broad academic skills were categorized as having “parental concern” (*n* = 12). Examples of these types of parent responses included “ADHD,” “reading issues,” and “not very verbal.” For the remaining participants (*n* = 59), parents either responded negatively to the question about concern or provided a response that was unrelated to attention, language, reading, or academics. Example responses included, “allergies,” “heart murmur,” and “speech therapy for R sounds.” By grouping attention, language, reading, and academic concerns, we created a potentially heterogeneous group of children with developmental concerns. However, this broad grouping was motivated by the literature connecting our constructs of interest (sustained selective attention and working memory) to disorders affecting all of these areas. In addition, parents may misattribute language difficulties to problems in another area (e.g., attention, reading, or general academics; Kamhi, 2004), and our brief contact with participants did not allow for us to conduct the diagnostic testing needed to differentiate among these disorders.

### Analyses

Data were preprocessed using custom-written Python scripts to calculate the dependent variables of interest for each of the two tasks. For the sustained selective attention task, participants' responses and reaction times were extracted from the E-Prime data file and the dependent measure *d’*_*adj*_ was calculated as described above. Similarly, for the visual working memory task, overall performance was computed by extracting the number of correct responses at each sequence length and calculating weighted accuracy as described above.

To support our argument that task scores provide a consistent estimate of performance, we calculated internal consistency reliability. For this analysis we implemented a two-pronged approach. First, we examined the internal consistency of each task by using the Spearman-Brown prediction formula to calculate the split-half correlation. This approach splits each participant's performance within the task into two data sets, as if the task had been given twice. Thus, if the tasks are consistently measuring the same construct there should be a high degree of correlation between each participant’s split data. To calculate the split-half reliability, we split each participant’s performance on a task into two sets of data using even- and odd-numbered trials. We then computed the corresponding dependent variables for both the odd and even trial sets for each participant. This yielded two dependent measure scores for each participant on each task. Using the Spearman-Brown prediction formula we calculated the correlation coefficient between the two dependent measure scores within each task to find the split-half reliability.

As a second approach we used bootstrap simulations to construct a confidence interval of split-half reliabilities by resampling participant data (Picheny et al., 2010; Pronk et al., 2022). This second approach was chosen to provide a confidence interval estimate for the calculated split-half reliability and to guard against over/underestimation due to confounds arising from trial structure and the splitting method of odd/even trials (Pronk et al., 2022). Our bootstrapping approach consisted of randomly sampling without replacement trials from each subject to generate simulated repetitions for each participant. We then used the same split-half calculation as above, computing first the dependent variable of interest for each participant within the task and then taking the correlation between the splits for each of the simulated repetitions. For our analysis we ran 1000 simulations of each participant's responses using the random sampling without replacement. Following this approach, we were able to estimate the sampling distribution of reliability scores for both tasks providing further evidence that the tasks consistently measure the same construct.

To support our argument that tasks are sensitive to a range of abilities within our intended age range, we examined score distributions visually and calculated descriptive statistics including range, skewness, and kurtosis. Skewness and kurtosis values were divided by their standard error to yield *z*-scores, indicating their deviation from 1. To examine potential floor and ceiling effects, we defined a ceiling score to be the highest possible task score (e.g., Gulledge et al., 2020). For the sustained selective attention task, the highest possible *d’*_*adj*_ score was 5.47 (achieved by detecting all 64 targets and ignoring all 256 distractors). For the working memory task, the highest possible score was 56 (achieved by answering all items correctly). We also defined a floor score based on the minimum potential score on the task. For the sustained selective attention task, we used a score of *d’*_*adj*_ ≤ 0, as a score of 0 represents an inability to discriminate between targets and distractors. For the working memory task, we used a score of 16 or less as this represents chance accuracy (50%) across 16 trials at the two-item sequence length. For each task, we were interested in the proportion of the sample at ceiling or floor (Gulledge et al., 2020). We adopted Gulledge et al.’s (2020) threshold of 15% or more of the sample at floor or ceiling as evidence of a problematic effect.

To address the arguments that the tasks measure separate but related constructs and that they measure skills that improve within our target age range of 4 to 10 years, we next calculated Pearson correlations between the two task scores, as well as between task scores and age. Because we expected age to affect scores on both tasks, we also calculated partial correlations controlling for age. Finally, to explore the tasks’ potential to index ability, we conducted point-biserial correlations between the binary measure of parent concern and task scores, controlling for age.

## Results

### Each task measures its intended construct

The design of both tasks within theoretically supported paradigms provides initial evidence of their validity (i.e., go/no-go for sustained selective attention and *n*-back plus complex span for working memory). As shown in Table 1, the two tasks are moderately correlated (*r*(68) = .48, *p* < *.001*). The relationship between tasks continues to hold after controlling for age (*r*(67) = .29, *p* = .016). Thus, performance on sustained selective attention and working memory is related, independent of age, although the association was weak.

**Table 1:** Correlations among age and cognitive tasks

	SSA	WM
Age	.494**	.529**
SSA	–	.474**
WM	.288*	–

Bivariate correlations are displayed above the diagonal, and partial correlations (controlling for age) are below the diagonal.

*SSA* sustained selective attention ability measured by *d’*_*adj*_ (d-prime with loglinear adjustment), *WM* working memory ability measured by weighted accuracy.

* *p* < .05, ** *p* < .01

### Task scores provide consistent estimates of a child’s sustained selective attention and working memory

We first calculated split-half reliability between even and odd trials, followed by bootstrapping simulations to generate a sampling distribution of split-half reliabilities. Table 2 displays the results of these calculations.

**Table 2:** Split-half reliability coefficients

	Reliability with even-odd split	Reliability from bootstrapping simulations		
		Mean	SD	Range
SSA	.942	.963	.007	.924–.988
WM	.916	.897	.019	.791–.962

*SSA* sustained selective attention, *WM* working memory.

As shown in Table 2, both reliability coefficients fall above .9 using a traditional (even-odd) splitting procedure, indicating that task performance is highly consistent across trials. Results of the bootstrapping simulations corroborate this by providing the mean, standard deviation, and range of reliability coefficients across 1000 simulations for each task.

### The tasks are sensitive to a range of abilities within the intended age range

Overall, the tasks were feasible for children in the 4- to 10-year age range, as evidenced by children’s ability to understand and complete the tasks. On the sustained selective attention task, all participants achieved sufficient accuracy in one practice phase to move on to the task. On the working memory task, 61 children advanced to the task after one practice phase and the remaining 10 children repeated the practice a second time before advancing. On both tasks, all children who began the tasks completed them.

**Fig. 4:**
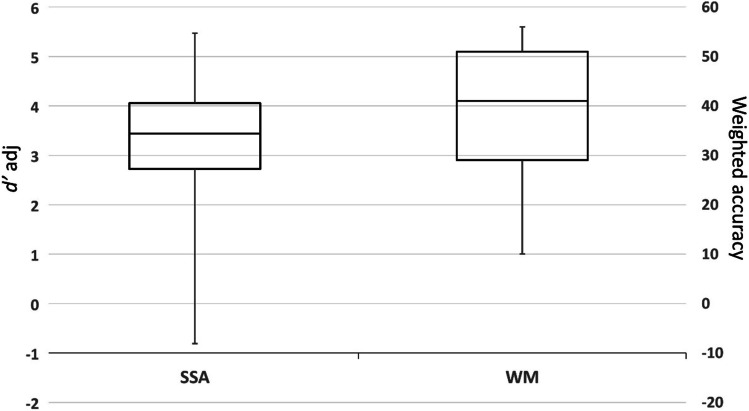
Box plots of score distributions for sustained selective attention and working memory tasks. SSA = sustained selective attention; plotted in *d’*_*adj*_ on the left vertical axis. WM = working memory; plotted in weighted accuracy on the right vertical axis

To more closely examine the distribution of scores within our target age range, we constructed box plots. Figure 4 displays these plots.

We also calculated descriptive statistics as well as the proportion of the sample at floor and ceiling. Table 3 displays these values for both tasks.

**Table 3:** Descriptive statistics for task outcomes

Measure	SSA	WM
M	3.28	37.69
SD	1.26	14.12
Range	−0.81–5.47	10–56
Skewness	−0.92 (0.29)	−0.48 (0.29)
Kurtosis	1.29 (0.57)	−1.01 (0.57)
Proportion of sample at floor	1/70 (1.4%)	10/71 (14.1%)
Proportion of sample at ceiling	3/70 (4.3%)	8/71 (11.3%)

Skewness and kurtosis are reported as value (standard error).

For both tasks, the scores obtained within the 4- to 10-year-old participant sample span the range of potential scores, with box plots and descriptive statistics supporting a continuous distribution. Although the theoretical range of *d’* is infinite, the practical range as implemented in this study is from 0 (indicating no ability to discriminate between targets and distractors) to 5.47 (a perfect rate for both targets and distractors). The sustained selective attention task scores span this range, with a mean near the center (*d’*_*adj*_ = 3.28). Transforming raw skewness and kurtosis values into *Z* scores yields −3.19 and 2.28, respectively, indicating a somewhat negatively skewed and heavy-tailed distribution. The box plot reinforces these findings.

On the working memory task, chance accuracy would result in a score of 16 and perfect accuracy yields a score of 56. Descriptive statistics again show a distribution across this range. The distribution is slightly negatively skewed (*Z*-score for skewness of −1.71) and light-tailed (*Z*-score for kurtosis of −1.78). Further evidence of the distribution of scores within the age range can be obtained by examining the proportion of the sample who performed well enough to advance to the next level of the task: 21% (15/71) did not advance beyond two-item sequences, 21% (15/71) advanced to three-item sequences, 28% (20/71) advanced to four-item sequences, and 30% (21/71) reached the highest, five-item sequence level.

For both tasks, the proportion of the sample scoring at floor or at ceiling remained below the threshold of 15% (Gulledge et al., 2020), supporting the argument that floor and ceiling effects fall within an acceptable range in the data. Proportions at floor or ceiling were higher for the working memory task than for the sustained selective attention task, likely due to the difference in number of trials across tasks (i.e., 16 working memory items and 320 sustained selective attention items).

### Performance on the tasks relate to external measures of development

The primary external measure of development in this study was age. The top row of Table 1 reports bivariate correlations between participant age and performance on the cognitive tasks. Participant age was positively related to performance on both cognitive tasks, indicating that older children had better performance than younger children.

The final exploratory analysis examined relations between broad parental concern and task performance. Age was not correlated with the binary measure of parent concern (*r*(69) = −.019, *p* =.877). Parent concern was negatively related to sustained selective attention performance after controlling for age (*r*(67) = -.343, *p* = .004), indicating that children whose parents expressed concern scored lower on the task than children whose parents expressed no concern. For working memory, there was no relation between parent concern and task performance after controlling for age (*r*(67) = −.087, *p* = .473).

## Discussion

We have described the design of two new tasks to measure children’s sustained selective attention and working memory using visual nonlinguistic stimuli. The sustained selective attention task presents spatial locations within a long and uninteresting task, following the CPT paradigm. The working memory task incorporates a key element of the *n*-back paradigm—determining if a complex stimulus matches the previous one—and also adapts stimulus length based on performance, consistent with the complex span paradigm.

The design of both tasks within existing paradigms for measuring sustained selective attention and working memory lends theoretical support that the new tasks created here also measure the same constructs. We examined further evidence of validity by calculating internal consistency reliabilities, distributions and floor and ceiling effects, and correlations between the tasks and with age. Overall, these analyses support task validity. Weak inter-task correlations provide evidence in favor of each task measuring related but distinct constructs (Swank & Mullen, 2017). Both tasks demonstrate high split-half reliability, using both a traditional even-odd split and a simulation of possible split-half reliabilities with random splits. This evidence indicates that scores can be generalized across the items in the tasks, which is necessary but not sufficient for validity arguments (Kane, 2013). For both tasks, children’s scores were distributed across the range of potential scores, with a somewhat negatively skewed distribution. Both tasks show evidence of sensitivity to development via correlations with chronological age.

For much of the evidence we examined here, the sustained selective attention task demonstrated stronger validity than the working memory task. Some of these advantages may relate to the length of the task. The sustained selective attention task has 320 trials versus just 16 for the working memory task. Tasks with more trials tend to have higher internal consistency reliability and may also avoid floor or ceiling effects more easily. However, increasing task length brings a tradeoff in terms of feasibility of administration. In addition, we were interested in measuring sustained selective attention and working memory separately. Increasing the length of the working memory task is likely to increase the degree to which sustained selective attention is required for task success, reducing the ability to measure working memory alone. The values we obtained for internal consistency reliability (.916) and floor and ceiling effects (14.1%, 11.3%) for the working memory task are sufficient, even if lower than those for sustained selective attention, and our decision to keep a shorter task length for working memory is supported by evidence of separation between results of the two tasks (*r(67)* = .288, with effects of age removed).

Finally, we conducted an exploratory analysis of the relation between task performance and a broad, dichotomous measure of parental concern. This analysis was limited by the nature of the parental concern measure and by the relatively small sample size. The environment in which these initial task validity data were collected precluded the type of in-depth testing needed to diagnose disorders of attention or language, and parent awareness of these difficulties may have been imperfect. We examined this relationship because the future intended use of the tasks is the identification of individual differences, including developmental disorders. The current data does not yet support this use, but does support further study.

### Extending the validity argument

Our initial validity argument focused on establishing the tasks as measures of their respective constructs, capable of detecting individual differences within the age range of interest. This initial study was limited by a modest sample size and a relatively brief study protocol totaling no more than 30 minutes, which did not include other measures of attention or memory. Ultimately, we envision one or both tasks contributing to the detection or identification of relatively subtle developmental disorders such as DLD and ADHD. Using the tasks to make inferences about the presence or absence of a disorder is a more ambitious interpretation of scores than simply detecting individual differences in ability and will require more extensive evidence of validity (Kane, 2013).

To extend the validity argument, it will be necessary to gather evidence within a sample of children who have confirmed developmental disorders: ADHD, DLD, or both. Cutpoints to determine acceptable performance on each task, likely by age group, should be set; the cutpoints can then be used to determine the diagnostic accuracy of each task (i.e., sensitivity, specificity, and likelihood ratios; e.g., Dollaghan, 2007). High diagnostic accuracy values would support the more ambitious argument that the tasks can be used in the identification process for each disorder. In addition, correlations between task performance and the severity of a developmental disorder would provide further evidence of task validity.

Given the overlap in underlying weaknesses between ADHD and DLD, task performance may need to be combined with other indicators of functional difficulty (such as short utterances or impulsive behaviors) to yield a specific diagnosis. We note, however, that such behavioral indicators alone are imperfect diagnostic indicators for either disorder, creating a need for measures of underlying processing weaknesses such as the ones created here. In the case of DLD in particular, variations in language-learning experiences (such as exposure to more than one language) complicate efforts to rely solely on behavioral indicators to identify the disorder (Ebert & Pham, 2019). The tasks described here are deliberately constructed using visual, nonlinguistic stimuli to reduce the effects of prior language-learning experiences and yield information about underlying processing abilities in linguistically diverse samples. Ultimately, this claim will require validation, meaning that the ability of the sustained selective attention and working memory tasks to identify disorder should be tested in groups of children with varied language-learning experiences (e.g., both monolingual and bilingual children).

## References

[CR1] Archibald LM, Gathercole SE (2006). Visuospatial immediate memory in specific language impairment. Journal of Speech, Language, and Hearing Research.

[CR2] Baddeley A (1992). Working memory. Science.

[CR3] Barry RJ, Clarke AR, McCarthy R, Selikowitz M, Rushby JA (2005). Arousal and activation in a continuous performance task: An exploration of state effects in normal children. Journal of Psychophysiology.

[CR4] Betts J, Mckay J, Maruff P, Anderson V (2006). The development of sustained attention in children: The effect of age and task load. Child Neuropsychology.

[CR5] Bishop DV, Snowling MJ, Thompson PA, Greenhalgh T, Adams C, Catalise-2 Consortium (2017). Phase 2 of CATALISE: A multinational and multidisciplinary Delphi consensus study of problems with language development: Terminology. Journal of Child Psychology and Psychiatry.

[CR6] Ciesielski KT, Lesnik PG, Savoy RL, Grant EP, Ahlfors SP (2006). Developmental neural networks in children performing a categorical N-Back task. Neuroimage.

[CR7] Conway AR, Kane MJ, Bunting MF, Hambrick DZ, Wilhelm O, Engle RW (2005). Working memory span tasks: A methodological review and user’s guide. Psychonomic Bulletin & Review.

[CR8] Corkum PV, Siegel LS (1993). Is the continuous performance task a valuable research tool for use with children with attention-deficit-hyperactivity disorder?. Journal of Child Psychology and Psychiatry.

[CR9] Cowan N (2016). Working memory maturation: Can we get at the essence of cognitive growth?. Perspectives on Psychological Science.

[CR10] Danahy K, Windsor J, Kohnert K (2007). Counting span and the identification of primary language impairment. International Journal of Language & Communication Disorders.

[CR11] Daneman M, Carpenter PA (1980). Individual differences in working memory and reading. Journal of Verbal Learning and Verbal Behavior.

[CR12] Dollaghan CA (2007). *The handbook for evidence-based practice in communication disorders*.

[CR13] Ebert KD, Kohnert K (2011). Sustained attention in children with primary language impairment: A meta-analysis. Journal of Speech, Language, and Hearing Research.

[CR14] Ebert KD, Pham G (2019). Including nonlinguistic processing tasks in the identification of developmental language disorder. American Journal of Speech-Language Pathology.

[CR15] Ebert KD, Rak D, Slawny CM, Fogg L (2019). Attention in bilingual children with developmental language disorder. Journal of Speech, Language, and Hearing Research.

[CR16] Gathercole SE, Pickering SJ, Ambridge B, Wearing H (2004). The structure of working memory from 4 to 15 years of age. Developmental Psychology.

[CR17] Gillam, R. B., Serang, S., Montgomery, J. W., & Evans, J. L. (2021). Cognitive processes related to memory capacity explain nearly all of the variance in language test performance in school-age children with and without developmental language disorder. *Frontiers in Psychology, 3990*. 10.3389/fpsyg.2021.724356 10.3389/fpsyg.2021.724356 PMC8490731 34621221

[CR18] Gomes H, Molholm S, Christodoulou C, Ritter W, Cowan N (2000). The development of auditory attention in children. Frontiers in Bioscience.

[CR19] Gordon R, Smith-Spark JH, Newton EJ, Henry LA (2020). Working memory and high-level cognition in children: An analysis of timing and accuracy in complex span tasks. Journal of Experimental Child Psychology.

[CR20] Greenberg, L.M., Holder, C., Kindschi, C.L., & Dupuy, T. (2020). T.O.V.a.® 9 clinical manual: Test of variables of attention continuous performance test. The TOVA company. http://files.tovatest.com/documentation/9/Clinical%20Manual.pdf

[CR21] Gulledge CM, Lizzio VA, Smith DG, Guo E, Makhni EC (2020). What are the floor and ceiling effects of patient-reported outcomes measurement information system computer adaptive test domains in orthopaedic patients? A systematic review. Arthroscopy: The Journal of Arthroscopic & Related Surgery.

[CR22] Hautus M (1995). Corrections for extreme proportions and their biasing effects on estimated values of *d′*. Behavior Research Methods, Instruments, & Computers.

[CR23] Heaton SC, Reader SK, Preston AS, Fennell EB, Puyana OE, Gill N, Johnson JH (2001). The test of everyday attention for children (TEA-Ch): Patterns of performance in children with ADHD and clinical controls. Child Neuropsychology.

[CR24] Huang-Pollock CL, Karalunas SL, Tam H, Moore AN (2012). Evaluating vigilance deficits in ADHD: A meta-analysis of CPT performance. Journal of Abnormal Psychology.

[CR25] Kamhi AG (2004). A meme’s eye view of speech-language pathology. Language, Speech, & Hearing Services in Schools.

[CR26] Kanaka N, Matsuda T, Tomimoto Y, Noda Y, Matsushima E, Matsuura M, Kojima T (2008). Measurement of development of cognitive and attention functions in children using continuous performance test. Psychiatry and Clinical Neurosciences.

[CR27] Kane MJ, Hambrick DZ, Tuholski SW, Wilhelm O, Payne TW, Engle RW (2004). The generality of working memory capacity: A latent-variable approach to verbal and visuospatial memory span and reasoning. Journal of Experimental Psychology: General.

[CR28] Kane MJ, Conway AR, Miura TK, Colflesh GJ (2007). Working memory, attention control, and the N-back task: A question of construct validity. Journal of Experimental Psychology: Learning, Memory, and Cognition.

[CR29] Kane MT (2013). Validating the interpretations and uses of test scores. Journal of Educational Measurement.

[CR30] Kane M, Lane S, Raymond MR, Haladyna TM (2015). Validation strategies: Delineating and validating proposed interpretations and uses of test scores. *Handbook of test development-2*^*nd*^*edition*.

[CR31] Karalunas SL, Gustafsson HC, Dieckmann NF, Tipsord J, Mitchell SH, Nigg JT (2017). Heterogeneity in development of aspects of working memory predicts longitudinal attention deficit hyperactivity disorder symptom change. Journal of Abnormal Psychology.

[CR32] Kaushanskaya M, Park JS, Gangopadhyay I, Davidson MM, Weismer SE (2017). The relationship between executive functions and language abilities in children: A latent variables approach. Journal of Speech, Language, and Hearing Research.

[CR33] Lavery MR, Bostic JD, Kruse L, Krupa EE, Carney MB (2020). Argumentation surrounding argument-based validation: A systematic review of validation methodology in peer-reviewed articles. Educational Measurement: Issues and Practice.

[CR34] Lin CC, Hsiao CK, Chen WJ (1999). Development of sustained attention assessed using the continuous performance test among children 6–15 years of age. Journal of Abnormal Child Psychology.

[CR35] Macmillan, N. A. & Creelman, C. D. (1991). *Detection theory: A user’s guide*. Cambridge University Press.

[CR36] Martinussen R, Hayden J, Hogg-Johnson S, Tannock R (2005). A meta-analysis of working memory impairments in children with attention-deficit/hyperactivity disorder. Journal of the American Academy of Child & Adolescent Psychiatry.

[CR37] Minnesota State Agricultural Society (2021). Minnesota State Fair attendance. https://www.mnstatefair.org/about-the-fair/attendance/. Accessed 10 Feb 2022

[CR38] Mirsky AF, Anthony BJ, Duncan CC, Ahearn MB, Kellam SG (1991). Analysis of the elements of attention: A neuropsychological approach. Neuropsychology Review.

[CR39] Miyake, A., & Shah, P. (1999). *Models of working memory*. Cambridge University Press.

[CR40] Pelegrina S, Lechuga MT, García-Madruga JA, Elosúa MR, Macizo P, Carreiras M, Fuentes LJ, Bajo MT (2015). Normative data on the n-back task for children and young adolescents. Frontiers in Psychology.

[CR41] Peng P, Fuchs D (2016). A meta-analysis of working memory deficits in children with learning difficulties: Is there a difference between verbal domain and numerical domain?. Journal of Learning Disabilities.

[CR42] Petersen SE, Posner MI (2012). The attention system of the human brain: 20 years after. Annual Review of Neuroscience.

[CR43] Picheny V, Kim NH, Haftka RT (2010). Application of bootstrap method in conservative estimation of reliability with limited samples. Structural and Multidisciplinary Optimization.

[CR44] Pronk T, Molenaar D, Wiers RW, Murre J (2022). Methods to split cognitive task data for estimating split-half reliability: A comprehensive review and systematic assessment. Psychonomic Bulletin & Review.

[CR45] Psychology Software Tools. (2018). *E-Prime 3.0* [Computer software]. https://pstnet.com/products/e-prime/

[CR46] Psychology Software Tools. (2019). *Chronos* [product information sheet]. https://pstnet.com/wp-content/uploads/2019/06/2019ProductSheetChronos.pdf.

[CR47] Riccio CA, Reynolds CR, Lowe P, Moore JJ (2002). The continuous performance test: A window on the neural substrates for attention?. Archives of Clinical Neuropsychology.

[CR48] Roebuck H, Freigang C, Barry JG (2016). Continuous performance tasks: Not just about sustaining attention. Journal of Speech Language and Hearing Research.

[CR49] Smolak E, McGregor KK, Arbisi-Kelm T, Eden N (2020). Sustained attention in developmental language disorder and its relation to working memory and language. Journal of Speech, Language, and Hearing Research.

[CR50] Swank JM, Mullen PR (2017). Evaluating evidence for conceptually related constructs using bivariate correlations. Measurement and Evaluation in Counseling and Development.

[CR51] Vugs B, Cuperus J, Hendriks M, Verhoeven L (2013). Visuospatial working memory in specific language impairment: A meta-analysis. Research in Developmental Disabilities.

[CR52] Yang HC, Gray S (2017). Executive function in preschoolers with primary language impairment. Journal of Speech, Language, and Hearing Research.

[CR53] Yaple Z, Arsalidou M (2018). N-back working memory task: Meta-analysis of normative fMRI studies with children. Child Development.

